# Endothelial Extracellular Vesicles—Promises and Challenges

**DOI:** 10.3389/fphys.2017.00275

**Published:** 2017-05-05

**Authors:** Carina Hromada, Severin Mühleder, Johannes Grillari, Heinz Redl, Wolfgang Holnthoner

**Affiliations:** ^1^AUVA Research Centre, Ludwig Boltzmann Institute for Experimental and Clinical TraumatologyVienna, Austria; ^2^Austrian Cluster for Tissue RegenerationVienna, Austria; ^3^Christian Doppler Laboratory on Biotechnology of Skin Aging, Department of Biotechnology, University of Natural Resources and Life SciencesVienna, Austria; ^4^Evercyte GmbHVienna, Austria

**Keywords:** extracellular vesicles, endothelial cells, exosomes, microparticles, pathology

## Abstract

Extracellular vesicles, including exosomes, microparticles, and apoptotic bodies, are phospholipid bilayer-enclosed vesicles that have once been considered as cell debris lacking biological functions. However, they have recently gained immense interest in the scientific community due to their role in intercellular communication, immunity, tissue regeneration as well as in the onset, and progression of various pathologic conditions. Extracellular vesicles of endothelial origin have been found to play a versatile role in the human body, since they are on the one hand known to contribute to cardiovascular diseases, but on the other hand have also been reported to promote endothelial cell survival. Hence, endothelial extracellular vesicles hold promising therapeutic potential to be used as a new tool to detect as well as treat a great number of diseases. This calls for clinically approved, standardized, and efficient isolation and characterization protocols to harvest and purify endothelial extracellular vesicles. However, such methods and techniques to fulfill stringent requirements for clinical trials have yet to be developed or are not harmonized internationally. In this review, recent advances and challenges in the field of endothelial extracellular vesicle research are discussed and current problems and limitations regarding isolation and characterization are pointed out.

## Introduction

Extracellular vesicles (EVs) are a heterogeneous population of phospholipid bilayer-enclosed vesicles that are secreted into the extracellular space by several cell types (Yáñez-Mó et al., 2015). Although once considered as cell debris lacking biological functions, EVs have recently become a focal point of interest in research with respect to their importance in the regulation of immune responses, contribution to the onset and progression of diverse pathologies such as age-associated diseases like neurodegenerative and cardiovascular diseases (CVDs), as well as their therapeutic potential (El Andaloussi et al., 2013; Weilner et al., 2013). EVs are commonly classified into three major subtypes based on vesicle biogenesis as well as size: exosomes, microparticles (MPs) or microvesicles, and apoptotic bodies. Ranging from approximately 30–100 nm in size, exosomes represent the smallest population among EVs. They are formed as intraluminal vesicles inside multivesicluar bodies (MVBs) in the endosomal compartment during the maturation of early into late endosomes (van der Pol et al., 2012; Weilner et al., 2013; Colombo et al., 2014). These MVBs subsequently either fuse with lysosomes to be degraded, or with the plasma membrane to be released as exosomes. The formation of MVBs is mostly mediated by the endosomal sorting complex required for transport (ESCRT) machinery, which consists of four complexes comprising approximately 30 proteins that overall sequester ubiquitinated transmembrane proteins in the endosomal membrane, and promote bud formation with sorted cargo and subsequent scission. However, MVB formation might also occur in an ESCRT-independent manner, e.g., via the tetraspanin CD63, the lipid metabolism enzymes sphingomyelinase, and phospholipase D2. Moreover, SNARE and Rab proteins (RAB7, RAB11, RAB27, and RAB35) seem to be involved in exosome secretion (Colombo et al., 2014). MPs, on the other hand, range between 100 and 1,000 nm and emerge directly from the outward budding and fission of the cell membrane (Combes et al., 1999; Heijnen et al., 1999; György et al., 2011; van der Pol et al., 2012). The formation of outward buds is driven by several membrane rearrangements due to increased Ca^2+^ levels: the enzymes flippase, floppase, and scramblase are recruited and activated to modify the lipid composition of the plasma membrane (i.e., the externalization of phosphatidylserine (PS), one major feature of MPs), and the protein calpain is furthermore activated to cleave cytoskeletal proteins to remodel the cytoskeleton. Additionally, also ARF6 and components of the ESCRT family have been implicated in the formation and release of MPs (Colombo et al., 2014; Minciacchi et al., 2015). The largest extracellular vesicles are apoptotic bodies released from dying cells and range from 1 to 5 μm in diameter (György et al., 2011; van der Pol et al., 2012).

The composition of EVs seems to be strongly influenced by the type and (patho) physiological condition of the secreting cell, the stimuli triggering their release, and the different pathways of EV biogenesis. Exosomes carry lipids, miRNAs, mRNAs, and proteins such as tetraspanins (CD9, CD63, and CD81), integrins, heat shock proteins (Hsp60, Hsp70, and Hsp90), ESCRT proteins (TSG101 and Alix), annexins, Rab proteins, GTPases, and flotillin (Mathivanan et al., 2010; van der Pol et al., 2012; Kourembanas, 2015). MPs also carry lipids (PS, cholesterol) and proteins including integrins, selectins, CD40L and MHC I and II (Safdar et al., 2016). Despite seemingly strong variations in size and features, there is still a demand to identify markers for distinguishing certain extracellular vesicle subpopulations in order to be able to truly understand the molecular mechanisms of biogenesis, secretion, and uptake as well as to assess the biological functions of the respective subtypes. In fact, there are several overlapping properties of exosomes and MPs that have led to the suggestion to collectively refer to them as “extracellular vesicles”: (i) size ranges cannot be considered absolute, (ii) lack of specific markers to uniquely identify a certain subtype, (iii) simultaneous release of all the different subtypes of EVs, and (iv) the impossibility to exclusively isolate pure fractions of a certain vesicle subtype from biological fluids or conditioned cell culture media (György et al., 2011; Gould and Raposo, 2013; Witwer et al., 2013). Therefore, the aim of this review is to discuss current problems regarding the isolation and characterization of EVs and summarize the versatile roles of endothelial extracellular vesicles in the human body as well as stimuli that trigger their release.

## Different cell types release extracellular vesicles of distinct functionality

Virtually all cell types are known to release EVs. Adiopose-, human umbilical cord- and bone marrow-derived mesenchymal stem cells (MSCs) have been reported to secrete cardioprotective (Lai et al., 2010; Arslan et al., 2013; Bian et al., 2014) and pro-angiogenic EVs (Bian et al., 2014; Chen et al., 2014; Zhang et al., 2015), which also promote myogenesis and osteogenesis both *in vitro* and *in vivo* (Lopatina et al., 2014; Nakamura et al., 2015; Kholia et al., 2016; Narayanan et al., 2016). Furthermore, MSC-derived EVs have also been shown to have an unclear role in tumor progression by either inhibiting (Bruno et al., 2013; Lee et al., 2013; Lopatina et al., 2016) or promoting (Zhu et al., 2012; Vallabhaneni et al., 2015; Lopatina et al., 2016) tumor growth through the transfer of miRNAs. Tumor cell-derived EVs themselves are also involved in tumor progression, metastasis, endothelial cell (EC) migration, and angiogenesis as well as in the escape from immune surveillance (Kim et al., 2002; Wysoczynski and Ratajczak, 2009; Grange et al., 2011; Marton et al., 2012). Apart from that, EVs derived from immune cells have also been shown to elicit immune responses: Dendritic cells and B cells, for example, release exosomes that carry MHC class II molecules and are consequently involved in antigen presentation to T cells (Raposo et al., 1996; Théry et al., 2002; Segura et al., 2005; Muntasell et al., 2007). Similarly, ECs release EVs that have different effects on tissue regeneration. While high levels of endothelial MPs (EMPs) seem to impair angiogenesis, physiological levels have a positive effect on the formation of capillary-like structures *in vitro* (Taraboletti et al., 2002; Mezentsev et al., 2005). Taken together, EVs are secreted from most cell types and are able to elicit different responses in other cell types. This is accomplished by internalization of EVs into recipient cells, thereby transporting EV cargo into the cell. These uptake mechanisms include endocytosis, fusion with the recipient cell's membrane or uptake via binding to the target cell's membrane (Maas et al., 2017). In ECs, EV uptake has been shown to be mediated via the interaction of EV surface proteins such as tetraspanins with membrane receptors of the recipient cell (Mulcahy et al., 2014). Tumor-derived EVs bearing Tspan8-CD49d complexes, for example, have been shown to be readily internalized by rat aortic ECs, thereby enhancing EC migration, proliferation and sprouting (Nazarenko et al., 2010). A role of tetraspanins in EV uptake by ECs has been further confirmed by the fact that Tspan8-α4 complex-bearing EVs were incorporated by rat aortic ECs, with intercellular adhesion molecule (ICAM)-1 being a major ligand (Rana et al., 2012). In general, EV uptake by ECs can have various consequences: Tumor exosomes, for examples, have been reported to transfer miRNAs when taken up by ECs, thereby contributing to angiogenesis (Zhuang et al., 2012; Umezu et al., 2013; Figliolini et al., 2014; Minciacchi et al., 2015; Ciardiello et al., 2016). Furthermore, large tumor-derived EVs called oncosomes have been shown to induce migration of mouse dermal and tumor ECs *in vivo* (Di Vizio et al., 2012; Ciardiello et al., 2016). Interestingly, retrotransposons were found to be enriched in tumor EVs, which can be transported to ECs, thereby potentially altering their genome (Balaj et al., 2011; Redzic et al., 2014). Apart from tumor cell EVs, ECs have also been shown to internalize miRNA-enriched EVs derived from macrophage/monocyte cells, which mediated target gene expression and EC function, as well as enhanced EC migration (Zhang et al., 2010; Redzic et al., 2014). Furthermore, hepatocyte-derived EVs cannot only be incorporated into ECs, but can also induce endothelial dysfunction, which was attributed to their arginase-activity (Royo et al., 2017). Also, it has been shown that EVs released from endometrium-derived MSCs transfer miR-21 into ECs, thereby exerting cardioprotective and proangiogenic effects (Wang et al., 2017).

## Isolation of extracellular vesicles

Although different EV subpopulations, their biogenesis, function, and cargo are an emerging topic of interest, we are still facing a lot of limitations that need to be resolved specifically with respect to isolation and characterization techniques. EV isolation techniques are currently based on filtration, density gradient centrifugation, ultracentrifugation, immunoaffinity techniques, size exclusion chromatography, and commercially available exosome precipitation kits (Witwer et al., 2013; Van Deun et al., 2014). Although immunoaffinity methods allow to specifically select EVs by the interaction of antibody-coated beads with surface proteins of particles, EV yield is often rather low due to the possibility that some markers might not be present on all particles (Tauro et al., 2012; Momen-Heravi et al., 2013; Witwer et al., 2013). Using filters with pore sizes down to 100 nm, filtration enables the separation of differently sized particles, although bearing the risk of obtaining quite impure fractions as a result of larger particles breaking down into smaller ones under filtration pressure (György et al., 2011; Witwer et al., 2013). The most widely used method for EV isolation, however, is differential centrifugation (Van Deun et al., 2014). Differential centrifugation is the only method by which larger volumes can be processed and consists of one or more low-speed centrifugation steps to remove cells, cell debris and larger apoptotic bodies. These initial debri-depletion steps are then followed by centrifugation at 10,000–20,000 × g to isolate MPs, and finally a high-speed centrifugation step at 100,000–120,000 × g to concentrate exosomes (Witwer et al., 2013; Cvjetkovic et al., 2014) D. G. Although an enrichment of distinct MP and exosome fractions is feasible, absolute separation of these two populations is not possible (Witwer et al., 2013). There are several parameters that influence the isolation efficiency including the g-force, temperature, centrifugation time and rotor type used. Since this information is lacking in many publications, reproducible results as well as comparisons between different studies are challenging. Pelleting efficiency of a given rotor can be described by the *k*-factor, which takes into account centrifugation velocity and rotor dimensions, with a lower *k*-factor indicating a better pelleting efficiency (Stephenson, 2003; Witwer et al., 2013; Cvjetkovic et al., 2014; Jeppesen et al., 2014). Furthermore, high-speed centrifugation can lead to contamination by protein and particle aggregates, urging the incorporation of density gradients (Momen-Heravi et al., 2013; Linares et al., 2015). Size exclusion chromatography is used to separate EVs by size by trapping small EVs in pores resulting in a prolonged flow through (Böing et al., 2014). This method allows a fast isolation of EVs void of protein and vesicle contaminants (Böing et al., 2014). Additionally, there are commercially available kits that advertise fast and easy EV isolation by precipitation (Momen-Heravi et al., 2013), although often leading to low purity and altered functionality of EVs (Van Deun et al., 2014; Gámez-Valero et al., 2016). Since purification of virus-like particles (VLPs) has come into focus for large-scale purification for potential therapeutic applications, and since EVs to some extent behave similar to VLPs (Steppert et al., 2016), it can also be imagined that these areas might be cross-talking for establishing the widely necessary and hoped-for standardized purification techniques. One method for the isolation of viruses makes use of polyethylene glycol (PEG) and can be adapted for purification of EVs. PEG precipitation is an inexpensive technique that allows easy and rapid isolation of EVs from large amounts of media (Rider et al., 2016). The benefits and disadvantages of the different techniques are summarized in Table 1.

**Table 1:** **Comparison of different EV isolation and characterization methods**.

**Technique**	**Advantages and disadvantages**
**ISOLATION OF EXTRACELLULAR VESICLES**	
Immunoaffinity techniques	+ antibody-specific selection of EVs − low yield (Tauro et al., 2012; Witwer et al., 2013)
Size exclusion chromatography	+ no co-isolation of protein and vesicle aggregates + quick − Not suitable for large volumes (Böing et al., 2014)
Filtration	+ separation of vesicles of different sizes − impure fractions: high pressure breaks larger EVs into smaller ones (György et al., 2011; Witwer et al., 2013)
Differential ultracentrifugation	+ enrichment of MPs and exosomes possible − many variations in g-forces and centrifugation times − co-isolation of contaminants (Witwer et al., 2013; Van Deun et al., 2014)
Density gradient ultracentrifugation	+ high purity possible + no confounding protein aggregates − labor-intensive (Van Deun et al., 2014)
Commercially available precipitation kits	+ no expensive equipment + easy to use − low purity − alters functionality of vesicles (Van Deun et al., 2014; Gámez-Valero et al., 2016)
PEG precipitation	+ inexpensive, easy and fast + sufficient amount of protein and RNA can be yielded for proteomics and sequencing analyses − high toxicity of PEG-derived EV preparations (Gámez-Valero et al., 2016; Rider et al., 2016)
**CHARACTERIZATION OF EXTRACELLULAR VESICLES**	
Electron microscopy	+ analysis of particle size and morphology − sample preparation time consuming − sample preparation might alter EV size and morphology − not suitable for quantitative analysis (György et al., 2011; Witwer et al., 2013; Mehdiani et al., 2015)
Western Blot	+ detection of EV-specific cargo and surface proteins − no quantitative analysis for EV number − large quantities of media required (Witwer et al., 2013)
Flow cytometry	+ quantitative analysis of particles + qualitative analysis of EVs by fluorescent labeling of specific surface markers − lower detection limit of flow cytometers: not suitable for exosomes − swarm effect (detection of multiple particles as one single event) − measurement of protein and antibody aggregates possible (Witwer et al., 2013; Mehdiani et al., 2015)
Nanoparticle tracking analysis	+ quantitative analysis of particles down to 30 nm + qualitative analysis of EVs by fluorescent labeling of specific surface markers − light scattering-based NTA does not allow qualitative analysis − fluorescence-based NTA requires large material quantities

## Characterization of endothelial extracellular vesicles

Standard techniques for quantification of EVs include optical methods such as electron microscopy, flow cytometry, and nanoparticle tracking analysis (NTA) in addition to non-optical techniques such as Western blotting. EMPs are characterized through the expression of various EC-specific surface markers, including CD31, CD54, CD62E, CD105, CD144, CD146, and von Willebrand factor (Dignat-George and Boulanger, 2011; Markiewicz et al., 2013). However, apart from CD62E and CD144, these markers are not exclusively expressed by ECs and hence, several markers need to be combined to assess the endothelial origin of MPs and exclude MPs of different origins, such as platelets (Dignat-George and Boulanger, 2011). Flow cytometry has the power to characterize EMPs by fluorescent labeling of these surface markers, albeit this method is limited for the detection of larger MP given the lower detection limit of flow cytometers. Moreover, particle determination is potentially confounded by protein aggregates (Witwer et al., 2013; Mehdiani et al., 2015). A promising complementary method to flow cytometry is NTA, which allows the characterization of particles as small as 30 nm. Particles are visualized by scattering of laser light. Based on Brownian motion, the average particle size is then calculated by the Stokes-Einstein equation, according to which a particle's size is in inverse proportion to its diffusion (Dragovic et al., 2011; Gardiner et al., 2013; Witwer et al., 2013; Mehdiani et al., 2015). As a semi-quantitative method, Western Blotting allows detection of EV-specific proteins independent of their size. However, the quantity of particles cannot be determined (Witwer et al., 2013). Electron microscopy, on the other hand, provides not only evidence for the presence of particles, but also the assessment of particle size and morphology. However, this method is unsuitable for the determination of particle concentration and moreover, sample preparation is time-consuming (Witwer et al., 2013; Mehdiani et al., 2015). Atomic force microscopy (AFM) allows three-dimensional imaging of EVs in aqueous fluids while at the same time preserving their state, with a resolution down to the nm scale (Harrison et al., 2014; Sebaihi et al., 2017). Additionally, there are some newer methods emerging in the field of EV analysis: Nanoscale fluorescence activated cell sorting (nanoFACS), for example, is a rapidly advancing and highly promising new method that allows both analysis and sorting of individual EVs as small as 40 nm (Brock et al., 2015; Jones, 2017). Imaging flow cytometry, on the other hand, combines the features of conventional flow cytometry with high-resolution imaging to allow the simultaneous and accurate quantification of both larger and smaller EVs down to 20 nm. These devices collect both image and fluorescence intensity data with a CCD camera, and enable the visualization of each individual particle that passes through the flow cell to additionally provide morphological confirmation (Headland et al., 2014; Clark, 2015; Erdbrügger and Lannigan, 2016). Superresolution microscopy (SRM) is able to exceed the diffraction limit of light, thereby allowing the imaging of structures down to 20–40 nm. Although SRM can visualize internalized EVs and thereby assess their localization inside target cells, distinguishing individual EVs remains difficult. Moreover, these methods are still limited by the lifetime of fluorochromes and the size of antibodies of approximately 15 nm (Araldi et al., 2012; Flynn and Yin, 2016). These novel methods might soon advance the field of EV research by providing optimized as well as more accurate analysis techniques.

As mentioned before, the establishment of standardized purification and characterization protocols would be of utmost importance for safe clinical application of EVs. The use of different analysis methods has been shown to greatly affect particle concentration, thereby rendering comparison of different characterization techniques highly challenging (Maas et al., 2015). For example, some of the previously considered classical exosome markers (i.e., flotillin, Hsp70) have also been shown to be present in larger EVs, thereby potentially questioning the reliability of previous data (Kowal et al., 2016). Given the great variety of isolation methods, EV purification, quality, and cargo greatly varies (Van Deun et al., 2017; Whiteside, 2017). The demand for these standardized protocols is, however, complicated by the fact that different cell lines produce different EVs that require different isolation parameters for optimal purification, as well as by the heterogeneous morphology and composition of EVs (Jeppesen et al., 2014; Szatanek et al., 2015; Erdbrügger and Lannigan, 2016). Nevertheless, the International Society of Extracellular Vesicles published a guideline including requirements necessary for sample collection, EV isolation and analysis to ease comparability of results (Witwer et al., 2013; Lötvall et al., 2014). Furthermore, the EV-TRACK knowledgebase (http://evtrack.org) collects methodological specifications from both published and unpublished experiments and has been established to promote standardization of EV research, provides researchers with relevant experimental parameters and facilitates interpretation of results (Van Deun et al., 2017).

## Endothelial extracellular vesicles

EMPs are released from ECs upon activation or apoptosis. Accounting for approximately 5–15%, EMPs constitute a large subclass of all circulating MPs in peripheral blood, albeit the majority of circulating plasma EVs are derived from platelets and erythrocytes, which together account for over 50% (Combes et al., 1999; Dignat-George and Boulanger, 2011; Markiewicz et al., 2013; Arraud et al., 2014). Although exerting various effects in the human body, EMPs are overall considered to impair the vascular function by being pro-coagulative (Combes et al., 1999) and pro-inflammatory (Buesing et al., 2011), as well as by mitigating nitric oxide (NO) production from ECs (Brodsky et al., 2004; Densmore et al., 2006).

**Figure 1:**
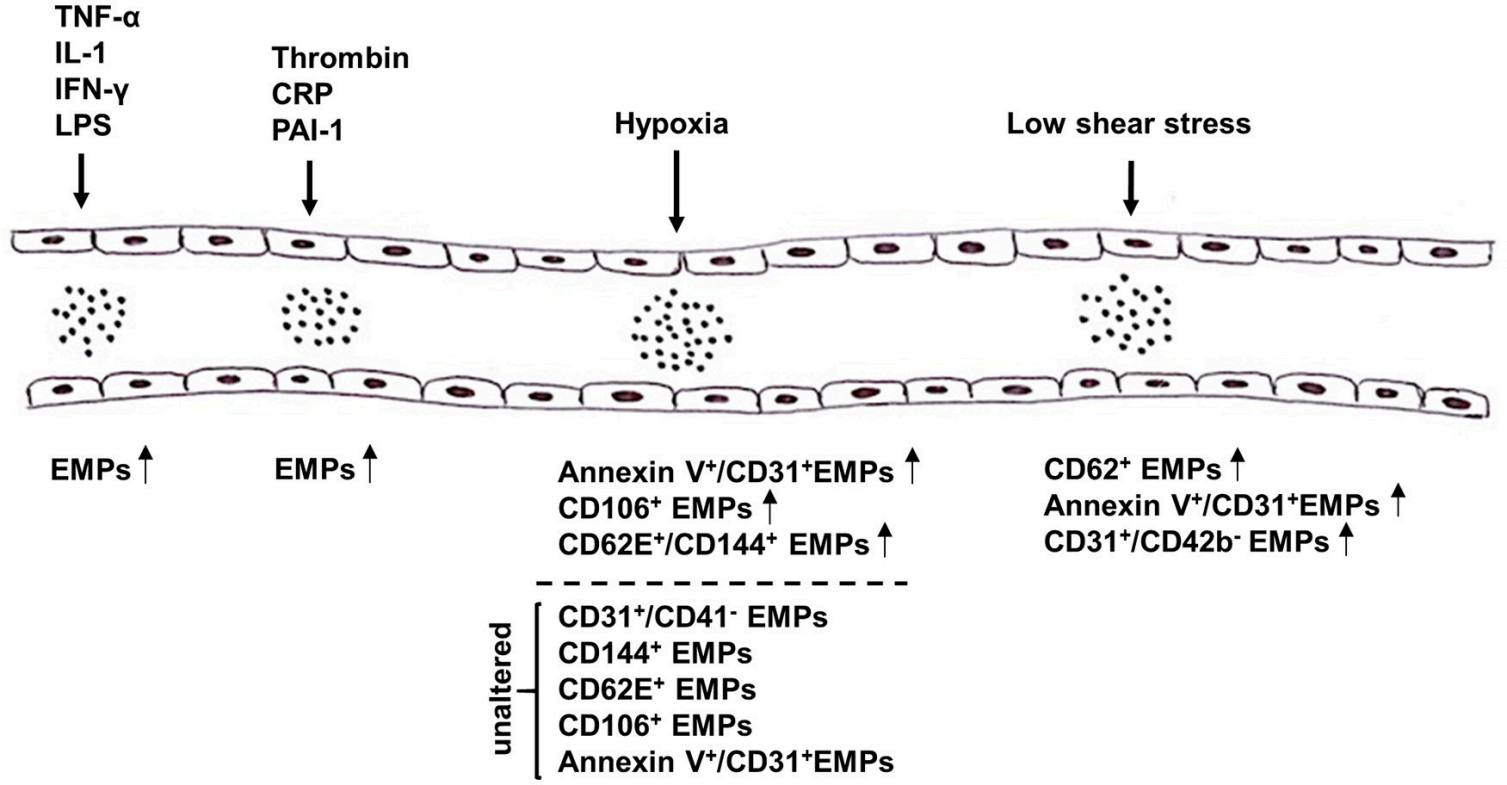
**Summary of triggers that mediate the release of microparticles (MPs) from endothelial cells (ECs)**. Release of endothelial microparticles (EMPs) into the circulation is induced in response to pro-inflammatory cytokines, e.g., TNF-α, IL-1, IFN-γ, and LPS, as well as thrombin, CRP and PAI-1. While high shear stress inhibits the release of EMPs, results on the effect of hypoxia on EMP release remain controversial. Several markers have been reported to be used alone or in combination to detect EMPs, e.g., AnnexinV, CD31, CD106, CD144, and CD62E. TNF-α, tumor necrosis factor α; IL-1, interleukin-1; IFN-γ, interferon-γ; LPS, lipopolysaccharide; CRP, C-reactive protein; PAI-1, plasminogen activator inhibitor-1.

Various studies reported the impact of certain stimuli on the release of EMPs from ECs both *in vitro* and *in vivo* (Figure 1), thereby not only providing insight into their contribution to the onset and progression of diseases, but also shedding light on novel therapeutic options. One of these triggers is the pro-inflammatory cytokine tumor necrosis factor-α (TNF-α) (Combes et al., 1999; Szotowski et al., 2007; Liu et al., 2013; Yamamoto et al., 2015), which induces endothelial activation, the consequence of which being a shift from a quiescent and protective to a pro-coagulant and vasoconstrictive state (Sumpio et al., 2002). Combes et al. were the first to show that stimulation of human endothelial cells from the umbilical vein (HUVEC) with TNF-α leads to a dose-dependent increase in the release of EMPs, which could be reversed after co-treatment with anti-TNF-α antibody, and furthermore observed an induction of tissue factor (TF) on the surface of these endothelial MPs (Combes et al., 1999). Proteomic analyses showed that secreted EMPs contain certain proteins that are also found in the originating ECs after TNF-α stimulation, and transfer of these proteins by EMPs could be an important mechanism in the interaction between EMPs and their target cells (Liu et al., 2013). Other inflammatory agents, including interleukin-1 (IL-1), interferon-γ (IFN-γ) and bacterial lipopolysaccharide (LPS) have also been shown to induce the release of EMPs, which were found to contain specific miRNAs that were either entirely absent or present in significantly lower amounts compared to EMPs derived from unstimulated ECs (Yamamoto et al., 2015). Furthermore, these miRNAs might be able to mediate inflammatory responses of ECs by mediating the gene expression profiles of pericytes (Yamamoto et al., 2015). Apart from pro-inflammatory cytokines, other agents such as thrombin (Sapet et al., 2006), C-reactive protein (CRP) (Wang et al., 2007), and plasminogen activator inhibitor-1 (PAI-1) (Brodsky et al., 2002) are also capable of inducing the release of MPs from ECs (Dignat-George and Boulanger, 2011).

In addition to pro-inflammatory agents, hypoxia has also been shown to alter the release of MPs from ECs, with highly controversial effects being reported (Vince et al., 2009; Ayers et al., 2014; Lichtenauer et al., 2015; Tuleta et al., 2015; Pichler Hefti et al., 2016). On the one hand, Lichtenauer et al. and Vince et al. found elevated levels of circulating AnnexinV^+^/CD31^+^ and CD106^+^ EMPs, respectively, in patients after exposure to temporary hypoxic conditions (Vince et al., 2009; Lichtenauer et al., 2015). On the other hand, Ayers et al. did not observe significant changes in the amount of circulating CD31^+^/CD41^−^, CD144^+^, CD62E^+^, and CD106^+^ EMPs *in vivo* after short-term hypoxic exposure (Ayers et al., 2014). Pichler Hefti et al. subjected healthy volunteers to hypobaric hypoxia and only found elevated levels of CD62E^+^/CD144^+^ EMPs, but not of AnnexinV^+^/CD31^+^ EMPs, indicating that endothelial dysfunction caused by hypoxia is induced by endothelial activation (Pichler Hefti et al., 2016). While these groups investigated the effects in healthy volunteers, Tuleta et al. assessed the effects of intermittent hypoxia in initial and advanced stages of vasculopathy in mice. Since elevated levels of AnnexinV^+^/CD31^+^ EMPs after hypoxic exposure were solely found during early but not advanced stages of vasculopathy, hypoxia might only impair endothelial dysfunction at early stages of vascular diseases but does worsen already advanced stages any further (Tuleta et al., 2015).

Under physiologic conditions, ECs are subjected to laminar shear stress (SS), which is responsible for EC survival and quiescence. Laminar SS is therefore required for maintaining normal vascular function by exerting anti-coagulant, anti-inflammatory, and vasodilatory effects through the release of NO (Boulanger et al., 2007; Vion et al., 2013). Concurrently, it has been shown that reduced SS, as for example caused by disturbed blood flow, impairs endothelial function by inducing apoptosis, morphological changes and the release of factors promoting platelet aggregation and vasoconstriction (Paszkowiak and Dardik, 2003). Kim et al. and Vion et al. investigated the effects of laminar SS on EMP release *in vitro* as well as *in vivo* and found significantly lower levels of circulating AnnexinV^+^/CD144^+^ and CD62E^+^ EMPs, respectively, after exposure to high SS compared to low SS (Vion et al., 2013; Kim et al., 2015). These alterations were seemingly caused by increased NO production induced by high levels of SS, which in turn hampered the secretion of EMPs (Vion et al., 2013). Moreover, two other groups were able to prove a strong correlation between laminar SS and the release of EMP levels *in vivo*. While Boulanger et al. assessed the impact of laminar SS on circulating EMPs in hemodialyzed end stage renal disease patients, Jenkins et al. were the first to examine the *in vivo* effects of disturbed blood flow on EMP release in healthy subjects (Boulanger et al., 2007; Jenkins et al., 2013). Patients suffering from end-stage renal disease are prone to have elevated levels of EMPs due to decreased SS, and hemodialysis induces an increase in brachial artery SS, which led to a significant decrease of these EMPs (Boulanger et al., 2007). Jenkins et al., on the other hand, promoted low SS in healthy volunteers by inducing a localized disturbed blood flow by using an occlusion cuff on the forearm, resulting in significantly increased levels of CD62E^+^ and CD31^+^/CD42b^−^ EMPs compared to the control arm (Jenkins et al., 2013).

In contrast to EMPs, endothelial exosome concentration as well as size was not influenced by stimuli such as hypoxia and TNF-α (de Jong et al., 2012). However, there are contradictory findings regarding changes in the exosome concentration with either unaltered (de Jong et al., 2012) or increased (Wu et al., 2016) exosome release upon stimulation with high glucose concentrations. Furthermore, hypoxia and TNF-α, but not high glucose concentrations, resulted in altered protein and RNA composition of endothelial exosomes, which reflected cellular stress conditions (de Jong et al., 2012). Hence, exosomes have gained interest as a source of biomarkers to assess the physiological condition of their cell of origin (de Jong et al., 2012). Both hypoxia and LPS have furthermore been shown to increase the release of exosomes from pulmonary artery ECs, which were involved in enhanced proliferation and resistance to apoptosis in pulmonary artery smooth muscle cells (Zhao et al., 2017). Additionally, ECs stimulated with transforming growth factor (TGF)-β1 induced shedding of VEGFR2-containing exosomes, which seemed to limit the effects of angiogenic stimuli on vascular sprouting (Jarad et al., 2017).

## Endothelial extracellular vesicles in diseases and their therapeutic potential

Although ECs constitutively secrete EVs into the blood in low concentrations under physiological conditions, endothelial EV levels have been found to be elevated in various diseases involving endothelial injury or dysfunction. For example, increased plasma levels of EMPs have been found in patients suffering from diabetes mellitus (Sabatier et al., 2002a; Koga et al., 2005; Tramontano et al., 2010; Jansen et al., 2013). Consequently, Jansen et al. showed that EMPs released from ECs cultured under high glucose conditions induced endothelial dysfunction, vascular inflammation, and promoted atherosclerosis *in vivo* (Jansen et al., 2013). Interestingly, there seems to be a toxic dose of EMPs isolated from quiescent ECs. Mezentsev et al., observed a significant impairment of angiogenesis, decrease in cell proliferation as well as an increase in apoptosis *in vitro* when treating cells with pathophysiological concentrations of 10^5^ EMPs/ml. Physiological concentrations of 10^3^ and 10^4^ EMPs/ml, however, did not significantly affect angiogenesis (Mezentsev et al., 2005). Further pathologies that implicate EMP-related endothelial dysfunction and injury include preeclampsia (Bretelle et al., 2003; González-Quintero et al., 2003, 2004; Petrozella et al., 2012), chronic renal failure (Faure et al., 2006), thrombotic thrombocytopenic purpura (TTP) (Jimenez et al., 2001), and multiple sclerosis (Minagar et al., 2001).

In CVDs specifically, elevated plasma levels of EMPs have been associated with acute coronary syndrome (Mallat et al., 2000; Bernal-Mizrachi et al., 2003), including myocardial infarction, angina pectoris, and myocardial ischemia, all of which are characterized by the accumulation of atherosclerotic plaques that finally lead to decreased blood flow to the heart (Kumar and Cannon, 2009). The pro-coagulant activity of EMPs is attributed to their expression of negatively charged PS and TF on the surface, which allows the interaction with coagulation factors and the activation of the extrinsic coagulation pathway, respectively (Combes et al., 1999; Abid Hussein et al., 2008; Dignat-George and Boulanger, 2011). While Combes et al. first showed that TNF-α stimulation triggers thrombin generation *in vitro* via the release of TF-exposing EMPs, Abid Hussein et al. showed that also IL-1α induced the secretion of these pro-coagulant EMPs, which were not only capable of inducing thrombin generation *in vitro* but also *in vivo* (Combes et al., 1999; Abid Hussein et al., 2008). Additionally, TF-bearing EMPs have been shown to bind to monocytes via the interaction of intercellular adhesion molecule (ICAM)-1 on EMPs and integrin on monocytes, thereby inducing a TF-dependent procoagulant activity in these cells (Sabatier et al., 2002b). Finally, sickle cell disease has been associated with increased levels of TF-exposing EMPs suggesting that there is a link to thrombotic events, such as stroke (Shet et al., 2003). Hence, TF- and PS-expressing, pro-coagulant EMPs contribute to the onset and progression of CVDs and thrombosis.

In contrast to their deleterious role, EMPs can also exert beneficial effects, such as promoting EC survival. For example, it has been shown that EMPs have the capacity to modulate the angiogenic properties of endothelial progenitor cells *in vitro* by inducing plasmin generation (Lacroix et al., 2007). Furthermore, also the release of matrix metalloproteinase-containing EMPs exerted a pro-angiogenic role *in vitro* (Taraboletti et al., 2002). However, tube formation was only induced in low numbers, whereas higher numbers decreased this pro-angiogenic capacity (Taraboletti et al., 2002; Mezentsev et al., 2005; Lacroix et al., 2007), which was partly attributed to excessive plasmin generation, leading to extracellular matrix degradation and apoptosis (Lacroix et al., 2007).

Taken together, these findings highlight the versatile role of EMPs in the human body as well as their importance as markers of disease (Mezentsev et al., 2005). Whether EMPs maintain vascular homeostasis or contribute to the onset and progression of CVDs might depend on their composition and the stimulus triggering their release (Peterson et al., 2008; Dignat-George and Boulanger, 2011).

In contrast to EMPs, the effects of exosomes secreted from ECs are not well explored yet. However, it has recently been shown that endothelial exosomes are capable of transferring miRNAs to tumor cells. In particular, exosomes contained miR-503, which diminished tumor cell proliferation and invasion *in vitro* (Bovy et al., 2015). It has also been shown that ECs secrete exosomes containing Delta-like 4 ligand, which they can pass to other ECs, thereby promoting angiogenesis via inhibition of Notch signaling (Sheldon et al., 2010). Additionally, high glucose culture of glomerular ECs not only led to increased levels of exosomes, but also activated glomerular mesangial cells and promoted diabetic nephropathy via transfer of TGF-β1 mRNA (Wu et al., 2016). Finally, increased exosome secretion by senescent human ECs has been shown to impair osteogenesis of human MSCs *in vitro* by transfer of its selective cargo: while miR-31 is overrepresented in senescent EC-derived exosomes and inhibitory to osteogenic differentiation (Weilner et al., 2016b), the osteogenesis-promoting protein galectin-3 is underrepresented in EC-derived exosomes (Weilner et al., 2016a). This suggests EC-derived EVs also to cross-talk within the bone marrow niche and to be involved in the pathogenesis of osteoporosis, as circulating miR-31 is also found to be high in individuals with osteoporotic fractures (Weilner et al., 2016b).

## Outlook and conclusion

The pathophysiological roles of EC-derived EVs and their cargo in CVDs, osteoporosis, cancer, and infectious and neurodegenerative diseases are becoming increasingly recognized, thereby elucidating the clinical potential of endothelial EVs for novel therapeutic options. Since EVs can efficiently deliver their cargo into recipient cells, they might soon be used as promising therapeutic agents to treat these diseases. This, however, highlights the urgent need of a thorough investigation of the exact uptake and targeting mechanisms of EVs. Furthermore, the potential beneficial effects of endothelial MPs and especially exosomes in tissue regeneration and wound healing still remain to be investigated. More sophisticated technologies could soon answer key questions like the cell type-specific origin of EVs, additional, yet to be defined subsets of EVs, or the role of EVs in developmental processes. Consequently, a deeper understanding of the complex role of these vesicles in the next few years is warranted, allowing the exploration of the numerous possible clinical applications of EVs.

## Author contributions

CH drafted the manuscript. SM, HR, JG, and WH have written parts of the manuscript. All authors approved the final version of the manuscript.

### Conflict of interest statement

JG is co-founder of Evercyte GmbH and TAmiRNA GmbH. All other authors declare that the research was conducted in the absence of any commercial or financial relationships that could be construed as a potential conflict of interest.
